# Uptake of silver nanoparticles by monocytic THP-1 cells depends on particle size and presence of serum proteins

**DOI:** 10.1007/s11051-016-3595-7

**Published:** 2016-09-22

**Authors:** Katja Kettler, Christina Giannakou, Wim H. de Jong, A. Jan Hendriks, Petra Krystek

**Affiliations:** 1 Department of Environmental Science, Radboud University Nijmegen, Nijmegen, The Netherlands; 2 National Institute for Public Health and the Environment (RIVM), P.O. Box 1, 3720 BA Bilthoven, The Netherlands; 3 Department of Toxicogenomics, Maastricht University, P.O. Box 616, 6200 MD Maastricht, The Netherlands; 4 Philips Innovation Services, HighTech Campus 7, 5656 AE Eindhoven, The Netherlands; 5 Institute for Environmental Studies (IVM), VU University, De Boelelaan 1087, 1081 HV Amsterdam, The Netherlands

**Keywords:** Nanoparticle, Uptake kinetics, Size dependence, Internal cellular concentration, Serum proteins, Biomedicine

## Abstract

Human health risks by silver nanoparticle (AgNP) exposure are likely to increase due to the increasing number of NP-containing products and demonstrated adverse effects in various cell lines. Unfortunately, results from (toxicity) studies are often based on exposure dose and are often measured only at a fixed time point. NP uptake kinetics and the time-dependent internal cellular concentration are often not considered. Macrophages are the first line of defense against invading foreign agents including NPs. How macrophages deal with the particles is essential for potential toxicity of the NPs. However, there is a considerable lack of uptake studies of particles in the nanometer range and macrophage-like cells. Therefore, uptake rates were determined over 24 h for three different AgNPs sizes (20, 50 and 75 nm) in medium with and without fetal calf serum. Non-toxic concentrations of 10 ng Ag/mL for monocytic THP-1 cells, representing realistic exposure concentration for short-term exposures, were chosen. The uptake of Ag was higher in medium without fetal calf serum and showed increasing uptake for decreasing NP sizes, both on NP mass and on number basis. Internal cellular concentrations reached roughly 32/10 %, 25/18 % and 21/15 % of the nominal concentration in the absence of fetal calf serum/with fetal calf serum for 20-, 50- and 75-nm NPs, respectively. Our research shows that uptake kinetics in macrophages differ for various NP sizes. To increase the understanding of the mechanism of NP toxicity in cells, the process of uptake (timing) should be considered.

**Electronic supplementary material:**

The online version of this article (doi:10.1007/s11051-016-3595-7) contains supplementary material, which is available to authorized users.

## Introduction

Nanoparticles (NPs) have dimensions from 1 up to 100 nm by common consensus (European Commission 2011). Their unique physical and chemical properties arouse high interest in industry regarding their use for consumer products and medical applications as previously described in more detail (Kettler et al. 2016). The same properties that make them interesting for industrial applications, especially their small size and high reactivity, raise serious concerns regarding a potential risk to human health and biota on the long term (Christensen et al. 2010) through exposure via one of the four exposure routes: injection, dermal and intestinal uptake, and inhalation, the latest being considered the most important especially during NP production and processing (Maynard and Kuempel 2005). Exposure to NPs and subsequent uptake have been shown to affect various cell lines, including phagocytizing cells like THP-1-derived macrophages (Foldbjerg et al. 2009; Haase et al. 2011; Park et al. 2011; Singh and Ramarao 2012). Unfortunately, uptake kinetics and time-dependent internal cellular concentrations are often neglected in experiments with measurements after a single fixed point in time and toxicity is related to exposure dose instead. Uptake is known to be influenced by NP properties such as size, as shown for various cell lines in vitro. Unfortunately, uptake has only been detected after a fixed time point in many studies (He et al. 2010; Jiang et al. 2008; Lu et al. 2009; Rejman et al. 2004; Wang et al. 2010).

Uptake of NPs into cells, after contact via one of the four exposure routes, occurs through two fundamental biological processes, namely pinocytosis and phagocytosis, both tailor-fit for their biological function. These fundamental biological mechanisms are responsible for the uptake of molecules from the environment. NPs have been shown to be able to enter cells via these two mechanisms as summarized by (Kettler et al. 2014). Pinocytosis, a mechanism usually employed by eukaryotic cells, such as 16HBE14o cells, to take up small essential nutrients (Conner and Schmid 2003), is described in more detail elsewhere (Kettler et al. 2016). The vesicles reach sizes of up to 80 nm in diameter (i.d.) for caveolae-mediated endocytosis or 120 nm i.d. for clathrin-mediated endocytosis. Phagocytosis on the other hand is a more specialized mechanism that can be found in unicellular organisms as well as in complex multicellular animals (Cosson and Soldati 2008; Rosales 2005). Phagocytosis as part of the immune defense in animals is special in the way that it is restricted to a few types of cells, such as monocytes, neutrophils and macrophages, to which THP-1 cells belong. Phagocytosis is a ligand-induced process, so especially the presence or absence of serum proteins has great effects on NP uptake as summarized previously (Kettler et al. 2014); the formed vesicles reach sizes between 0.5 µm and 10 µm (Watts and Marsh 1992). In the presence of proteins, a so-called protein corona on the NPs surface may be formed, leading to uptake that can be increased, decreased or remained the same, depending on the surface composition of the NPs, the type of cell and proteins and/or other biomolecules present on the NP surface (Ikada and Tabata 1986; Nagayama et al. 2007; Sbarra and Karnovsky 1959).

Very few efforts have been made to study the size-dependent uptake of NPs with diameters smaller than 100 nm into phagocytizing cells. Uptake rates over time were determined in two studies only (Chithrani et al. 2006; Yu et al. 2012), while the majority only reports uptake at one single point in time (Lu et al. 2009; Tsai et al. 2012; Vonarbourg et al. 2006; Walkey et al. 2011). In addition, these studies are also inconclusive about the effect of size on uptake into phagocytizing cells. To overcome this lack of data, we determine uptake rates for different AgNPs sizes in cell culture medium with and without FCS in this study. Previously it was shown that similar AgNPs induced cellular toxicity for the murine macrophage cell line RAW264.7 at concentrations of approximately 10 µg/mL and higher (Park et al. 2011). In this study, low non-toxic concentrations of 0.01 µg/mL were used to study cell uptake without inducing toxicity. Cells were, therefore, exposed to NPs for different periods of time over 24 h under realistic concentrations for short-term exposures. These data will deliver new insights into the effect of NP size on their uptake rate and are required to model and predict NP uptake rates based on easily measurable NP properties. The number of applications and produced quantities of NPs are increasing (Hendren et al. 2013; Nowack and Bucheli 2007; Vance et al. 2015), and especially the combination of empirical data like ours and modeling will allow for time- and cost-effective risk assessment of the ever increasing number of NPs.

## Materials and methods

A detailed description of Materials and methods can be found elsewhere (Kettler et al. 2016), so here only a brief description is given.

### Nanoparticles

Keeping the possible vesicles size of up to 80 nm in diameter (i.d.) for caveolae-mediated endocytosis in mind and to avoid an overlap in the size distribution of NPs, we chose NPs with diameter of 20, 50 and 75 nm. The following AgNPs were purchased from NanoComposix Inc (San Diego, CA): 20 nm Citrate BioPure™ Silver, 50 nm Citrate BioPure™ Silver and 75 nm Citrate BioPure™ Silver. The supplier provided detailed information about the NPs characteristics (Kettler et al. 2016).

### Preparation of AgNPs dispersions

Serum proteins present in cell culture growth medium may have significant effects on uptake (Ikada and Tabata 1986; Nagayama et al. 2007; Sbarra and Karnovsky 1959). Therefore, these uptake studies were conducted with media with fetal calf serum (+FCS) and without (w/o). The dilutions of AgNPs dispersions were performed in complete cell culture medium (see below), either without or with FCS, prior to exposure. Final exposure concentrations of 0.01 µg Ag/mL were obtained in several steps of pre-dilutions. We used very low concentrations of 0.01 µg/mL at which we do not expect cellular cytotoxicity. Similar AgNPs (20 and 80 nm) of the same supplier induced cellular toxicity for the murine macrophage cell line RAW264.7 at concentrations of approximately 10 µg/mL and higher (Park et al. 2011). Also for THP-1 cells at low concentrations, no cytotoxicity was noted for similar (20 and 50 nm) AgNPs of the same manufacturer (H.M. Braakhuis, personal communication). In addition, these concentrations represent realistic concentrations for short-term occupational alveolar lung exposures (Gangwal et al. 2011).

### Cells and cell culture conditions

The human monocytic cell line (THP-1, ATCC) was used in this study. THP-1 cells were cultured in Roswell Park Memorial Institute (RPMI) 1640 cell culture growth medium, supplemented with 10 % fetal calf serum and antibiotics (1 % penicillin–streptomycin (Pen/Strep), all obtained from Gibco, The Netherlands). This is designated complete medium. Cells were subcultured usually twice a week not to exceed a concentration of 1 × 10^6^ viable cells/mL. Subcultures were started with a cell density of 2 × 10^5^ to 4 × 10^5^ viable cells/mL. Cells were constantly incubated at 37 °C and 5 % CO_2_ atmosphere. Seven days prior to exposure to AgNPs, THP-1 were differentiated to macrophages according to the following procedure: THP-1 were diluted to a concentration of 5 × 10^5^ viable cells/mL to which phorbol myristate acetate (PMA, Sigma, The Netherlands) was added to reach a final concentration of 30 ng PMA/mL. Afterward, 1 × 10^5^ viable cells were added per well of a 96-well flat-bottomed cell culture plate to differentiate and become adherent. On day 5, medium was replaced by fresh medium; on day 7 the cells were exposed to AgNPs as described in section “cellular uptake of AgNPs.”

### Cellular uptake of AgNPs

Cells were seeded in 96-well cell culture plates as described in the “cell and cell culture conditions”-section and exposed to 200 µl of 0.01 µg/mL AgNP dispersions. The total exposure times were 0, 2, 4, 8, 12 and 24 h. Note: Due to logistic aspects, the time points at which the samples have been obtained are slightly shifted sometimes (maximum 2 h). At the end of exposure, cells were thoroughly washed with Dulbecco’s phosphate-buffered saline (DPBS, without calcium, magnesium, phenol red, Gibco, the Netherlands) twice to remove loosely attached Ag ions and/or NPs from the cell membrane. 200 µl trypsin solution (Gibco, The Netherlands) was added to detach the cells from the bottom of the well. Through pipetting, it was made sure that all cells were detached, and successful detachment was confirmed by optical inspection with a light microscope (magnification 50×). Each experiment was conducted independently two times for 20-nm AgNPs, and three times for 50- and 75-nm AgNPs.

### Statistical evaluation and calculations

For the determination of uptake rate constants c and elimination rate constants k, experimental results were evaluated with Microsoft Excel 2010 and the corresponding application “Solver” according to the one compartment model using Eq. 1. Solver is a tool that determines the optimal value for variables, here c and k, within given limits in order to minimize differences between experimental and model data.

1 $$c\left( t \right) = \frac{c}{k} \times \left( {1 - e^{ - k \cdot t} } \right)$$

where t is the time of exposure in hours; c is the uptake rate constant in ng Ag·well^−1^ day^−1^; and k is the elimination rate constant in ng Ag·well^−1^ day^−1^. Values for k were set to a minimum of 10^−8^ in order to avoid divisions by zero during the calculations, and no other limits are set. Ag concentrations were standardized to a starting concentration of zero by subtracting the initial concentration in the cells from the concentrations in the cells at the later time point.

## Results

**Fig. 1:**
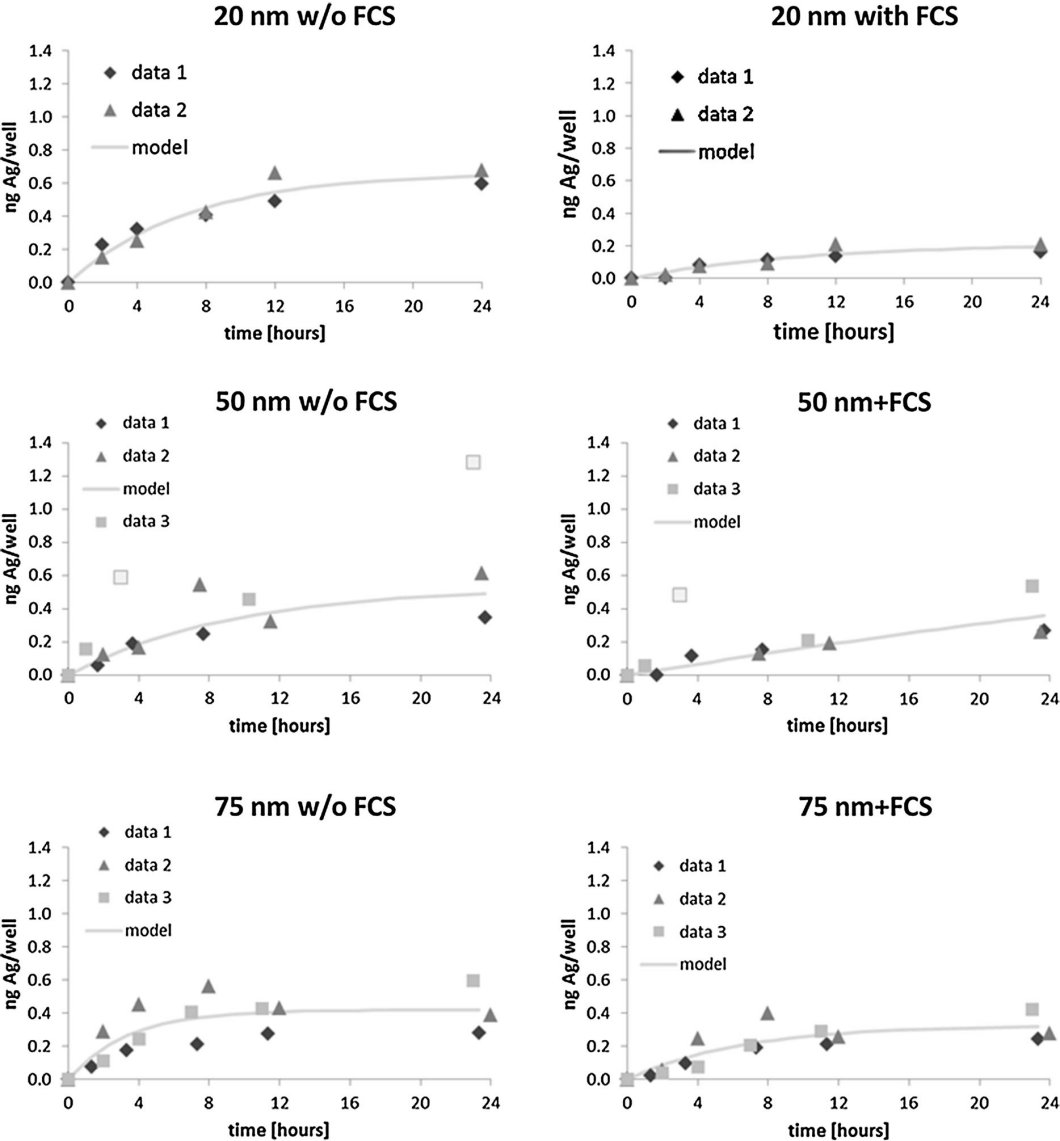
Ag amount in cells on mass basis with the fitted line for three NP sizes and medium without and with fetal calf serum based on at least two independent experiments (data 1 to data 3). The *light gray* data points belonging to data set 3 are indicated as outliers and were not considered in the determination of the fitted line. *Note* Due to logistic aspects, the time points at which the samples have been obtained are slightly shifted sometimes

The results for each replicate, as well as the according model uptake curves, for the three NP sizes are given in Fig. 1. The data points displayed in light gray belonging to data set 3 of the 50-nm samples w/o and +FCS were evaluated as outliers, likely due to insufficient washing, and were not considered in the calculations for the model curve. Uptake clearly levelled off during the time course for all samples except for the 50 nm +FCS where the rate of uptake stayed constant over 24 h. In our setup, the average uptake in the absence of FCS was higher than in the presence of FCS for 20- and 50-nm particles, reaching highest intracellular concentrations after 24 h. This effect was most pronounced for 20-nm particles reaching an approximately threefold higher maximum concentration after 24 h of exposure in the absence of FCS than in the presence of it. Average internal cellular concentrations after 24 h decreased with increasing particle size in the absence of FCS, reaching approximately 32, 25 and 21 % of the nominal concentration for 20-, 50- and 75-nm particles, respectively.

The uptake and elimination rates on mass basis are given in Table 1 and Table S1. The uptake rate constants are significantly different between media composition for 20- and 50-nm particles. In the absence of FCS, uptake was also faster for the smallest particles compared to 50-nm particles as represented by the significantly different 95 % confidence interval of the uptake rate constant c. No significant difference in uptake rate was found between the other NP sizes due to the large difference in uptake rates between replicates of 75-nm particles.

**Table 1:** Overview of the uptake rates based on mass [ng] Ag

NP size [nm], medium type	*c*	SD of *c*	95 % CI of *c*
20 w/o FCS	1.0 × 10^−1^	1.5 × 10^−2^	8.0 × 10^−2^–1.2 × 10^−1^
50 w/o FCS	5.6 × 10^−2^	2.0 × 10^−2^	3.3 × 10^−2^–7.8 × 10^−2^
75 w/o FCS	1.4 × 10^−1^	1.2 × 10^−1^	9.5 × 10^−3^–2.7 × 10^−1^
20 + FCS	2.1 × 10^−2^	1.1 × 10^−3^	1.9 × 10^−2^–2.3 × 10^−2^
50 + FCS	2.2 × 10^−2^	5.8 × 10^−3^	1.6 × 10^−2^–2.9 × 10^−2^
75 + FCS	5.6 × 10^−2^	3.5 × 10^−2^	1.6 × 10^−2^–9.6 × 10^−2^

Average uptake rate constants *c* and elimination rate constants *k*, their standard deviation (SD) and 95 % confidence interval (CI) based on mass, all given in ng Ag well^−1^ day^−1^

**Fig. 2:**
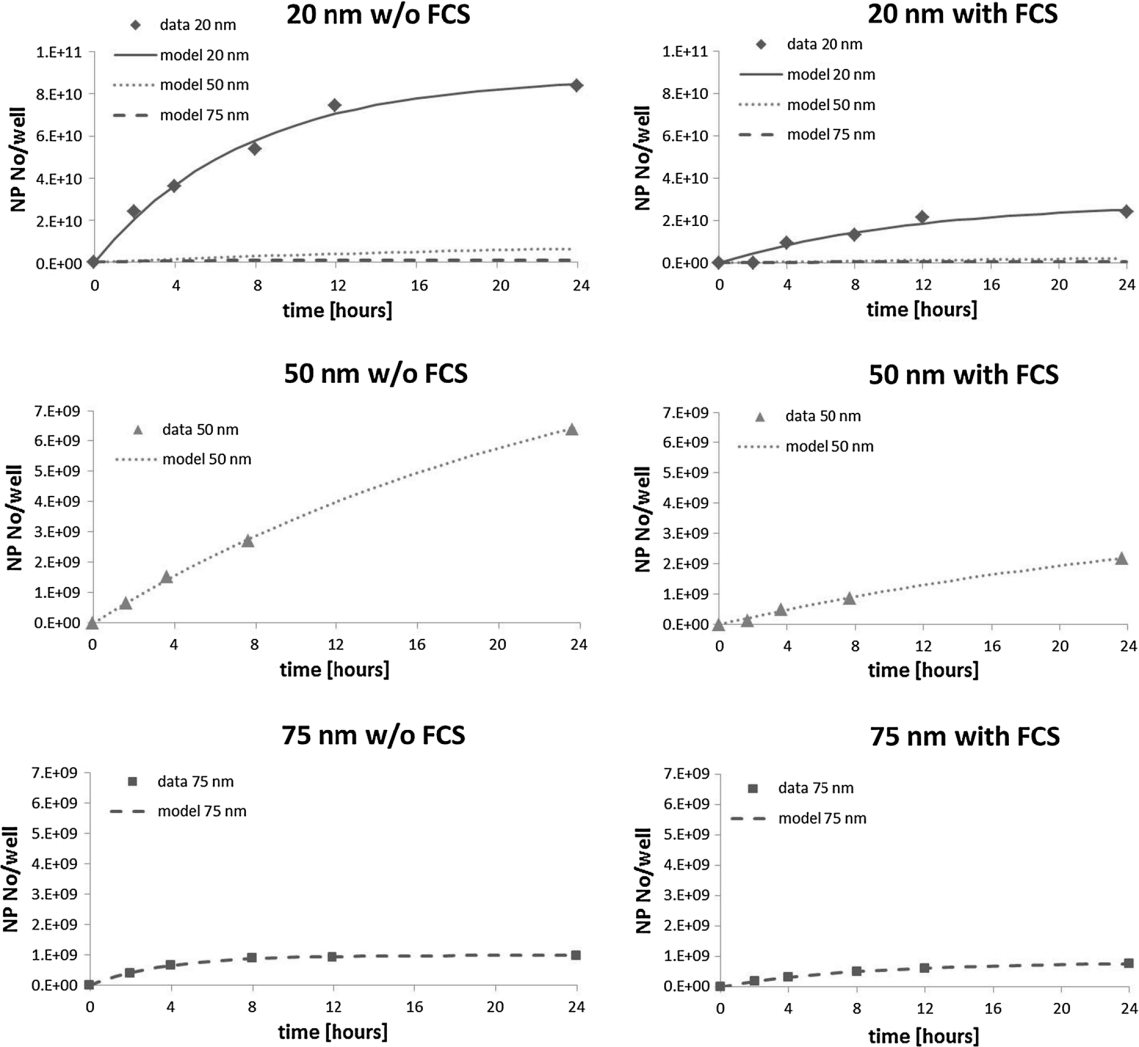
Average Ag content in cells on a NP number basis calculated from the independent mass-based values for three NP sizes and medium without and with fetal calf serum as shown in Fig. 1. The two *top* graphs show model data for all three NP sizes for easy comparison and calculated average NP numbers for 20-nm AgNPs. The four graphs on the *bottom* show the average Ag content and the corresponding model date for 50- and 75-nm AgNPs in more detail. *Note* Due to logistic aspects, the time points at which the samples have been obtained are slightly shifted sometimes, different scaling on the *y-axes*

The measurand in which results are presented (mass versus number of NP) is crucial because a size optimum based on mass does not necessarily lead to the same uptake optimum regarding NP numbers (Lévy et al. 2010). In our case, where uptake on mass basis is highest for the smallest NP size, the same will hold true on NP basis. Yet, when expressed on NP number basis, the difference might become significant. To test whether this is the case, and for comparison with other studies, NP numbers were calculated. The results without FCS change in so far that the uptake rate of 20-nm AgNPs differs significantly from those of 50- and 75-nm AgNPs. The uptake rates are not significantly different for 50- and 75-nm AgNPs in the absence of FCS. THP-1 cells favor the uptake of smaller AgNPs on NP number basis in the absence of FCS. This is depicted in Fig. 2.

## Discussion

Our results show that the cell culture medium composition in terms of the presence or absence of FCS affected the AgNP uptake in the macrophages considerably for 20-nm AgNPs (three times higher uptake) and 50-nm AgNPs, as confirmed by the literature (Ikada and Tabata 1986; Nagayama et al. 2007; Sbarra and Karnovsky 1959). In medium with FCS, a so-called protein corona around the AgNPs might be formed reducing uptake. The protein corona can lead to repulsive interaction with the negatively charged cell membrane as shown for anionic bovine serum albumin (Zhu et al. 2012). Yet, further confirmation for NPs of different charge and various types of serum proteins and other biomolecules that might be part of the protein corona is needed. When no FCS was present, final concentrations after 24 h were found to be higher for the smaller NPs. Uptake changes over time and levels off after approximately 12 h. Overall, our results show that uptake kinetics may differ per NP and therefore should be taken into account in toxicity studies (Table 2).

**Table 2:** Overview of the uptake rates based on AgNP numbers

NP size [nm], medium type	*c*	SD of *c*	95 % CI of *c*
20 w/o FCS	1.2 × 10^10^	1.6 × 10^9^	1.0 × 10^10^–1.5 × 10^10^
50 w/o FCS	4.5 × 10^8^	1.1 × 10^8^	3.4 × 10^8^–5.7 × 10^8^
75 w/o FCS	3.1 × 10^8^	2.6 × 10^8^	1.8 × 10^7^–6.0 × 10^8^
20 + FCS	2.5 × 10^9^	1.4 × 10^8^	2.3 × 10^9^–2.7 × 10^9^
50 + FCS	1.3 × 10^8^	3.4 × 10^7^	9.5 × 10^7^–1.7 × 10^8^
75 + FCS	1.2 × 10^8^	8.0 × 10^7^	3.3 × 10^7^–2.1 × 10^8^

Average uptake rate constants *c* and elimination rate constants *k*, their standard deviation (SD) and 95 % confidence interval (CI) based on AgNP numbers, all given in AgNPs well^−1^ day^−1^

After inhalation, lung macrophages are the most important cells for removal of the inhaled NPs from the lung. However, studies determining uptake by phagocytizing cells for particles in the nanorange and considering uptake over time are scarce and up to now inconclusive. Our results show that the amount taken up was in the order 20 > 50 = 75 nm without FCS and 50 = 75 > 20 nm in the presence of FCS on mass basis. In contrast to our findings that uptake is highest for smallest NPs, uptake by macrophages based on mass is less effective for smaller PEGylated NPs than for their larger counterparts (Yu et al. 2012). Uptake efficiency increases from 30-nm NPs, over 40-nm NPs, to 100-nm NPs. HeLa cells on the other hand favor the intermediate size with uptake in the following order: 50 > 30 > 74 > 14 > 100 on NP number basis (Chithrani et al. 2006). It would be interesting to see whether and how these trends change when expressed on mass basis.

We therefore recommend that the optimum uptake is presented in both measurands to allow for comparability between studies. Alternatively, data that allow for such conversions might be given. Such a conversion is difficult if, e.g., the total diameter is known but not the thickness of a coating. Levy et al. also showed that an optimum based on the number of NPs per cell does not necessarily lead to the same optimum in terms of mass (Lévy et al. 2010). Yue et al. graphically visualized the dependence of the size optimum on the measurand (number of particles per cell, particle volume per cell and particle surface area per cell) (Yue et al. 2010).

Different trends in the optimal particle size for uptake can be attributed to the numerous experimental factors that differ between studies, for example different NP materials and the use of FCS during incubation and whether opsonization takes place, as summarized previously by us (Kettler et al. 2014). Surface charge also affects NP uptake, and an increase in the absolute zeta potential usually leads to increased NP uptake in comparison with less charged NPs (Kettler et al. 2014). For the 50-nm AgNPs used in this study, charge might to some extend also determine uptake in comparison with the other AgNPs because their zeta potential differs from that of the 20- and 75-nm AgNPs from the same manufacturer. This stresses the need for commercially available NPs with only one property changed at a time or, ideally, that reference materials we made available.

Other factors that influence uptake are the use of various cell lines, different possible uptake mechanisms and exposure concentration. While phagocytosis is usually considered to take place for materials with sizes larger than 0.5 µm, Kuhn et al. showed that polystyrene NPs with sizes of 28 nm were taken up by J774A.1 macrophages via micropinocytosis and phagocytosis, as well as clathrin-mediated endocytosis (Kuhn et al. 2014). The exact uptake mechanism for AgNPs into THP-1 cells used in our experiments is unknown. The uptake mechanism can vary between differentiated and undifferentiated THP-1 cells (Lunov et al. 2011). In addition, the determination of the exact concentration and incubation time for the various inhibitors is very complex and has to be adjusted for each cell line separately (Kuhn et al. 2014). Saturation of uptake is observed; therefore, pinocytosis, a non-saturable process, as the uptake mechanism can be excluded (Fröhlich 2012).

## Conclusions

Our study clearly shows that the presence of FCS reduces cellular uptake for AgNPs of different sizes in the macrophage cell line THP-1 and that uptake should be reported both as NP number and as mass-based uptake. Our results show that reporting both in every study is important as the order of uptake in relation to NP size might change with the measurand and would be advantageous for easy comparison of results. Studies that determine uptake kinetics of particles with sizes below 100 nm by phagocytizing cells are scarce, stressing the need for further experimental investigation similar to the presented work. Therefore, patterns for the effect of particle size on uptake rate into phagocytizing cells are uncertain. Future developments will benefit most from studies where either the same NPs are tested in various phagocytizing cell lines known for their different endocytic uptake mechanisms (e.g., clathrin- versus caveolae-mediated endocytosis) or NPs of different chemical composition are tested in the same cell line in order to fill knowledge gaps and resolve apparent contradictions. Once patterns emerge, extrapolation between different NPs and between different experimental conditions will be possible. Modeling will be an indispensable tool to cover the large and continuously growing number of engineered NPs and to predict the uptake of untested NPs.

## Electronic supplementary material

Below is the link to the electronic supplementary material.

Supplementary material 1 (DOCX 21 kb)
