# Supplementary material for: Uptake of silver nanoparticles by monocytic THP-1 cells depends on particle size and presence of serum proteins
Source: J Nanopart Res. 2016 Sep 22;18(9):286. doi: 10.1007/s11051-016-3595-7 (PMC5034003; doi:10.1007/s11051-016-3595-7)
Supplement: Supplementary file 1 — Supplementary material 1 (DOCX 21 kb) [file 11051_2016_3595_MOESM1_ESM.docx]

**Journal of Nanoparticle Research**

**Uptake of silver nanoparticles by monocytic THP-1 cells depends on particle size and presence of serum proteins**

Katja Kettler^1*^, Christina Giannakou^2,3^, Wim H. de Jong^2^, A. Jan Hendriks^1^, Petra Krystek^4,5^

^1^ Department of Environmental Science, Radboud University Nijmegen, Nijmegen, The Netherlands; *telephone: + 31-(0)24 36 52 393, fax: +31-(0)24-355 34 50, e-mail address: [K.Kettler@science.ru.nl](mailto:K.Kettler@science.ru.nl)

^2^National Institute for Public Health and the Environment (RIVM), P.O. Box 1, 3720 BA Bilthoven, The Netherlands

^3^Department of Toxicogenomics, Maastricht University, PO Box 616, 6200 MD Maastricht, The Netherlands

^4^ former: Philips Innovation Services, High Tech Campus 7, 5656 AE Eindhoven, The Netherlands

^5^Institute for Environmental Studies (IVM), VU University, De Boelelaan 1087, 1081 HV Amsterdam, The Netherlands

**Table S 1 Overview of the elimination rates based on mass [ng] Ag**.

Average elimination rate constants k, their standard deviation (Std. dev) and 95 % confidence Interval (CI) based on mass, all given in ng Ag·well^-1^·day^-1^.

| NP size [nm], medium type | k | Std. dev. of k | 95% CI of k |
| --- | --- | --- | --- |
| 20 w/o FCS | 1.6·10^-1^ | 5.3·10^-2^ | 8.3·10^-2^-2.3·10^-1^ |
| 50 w/o FCS | 9.4·10^-2^ | 8.5·10^-2^ | -1.9·10^-3^-1.9·10^-1^ |
| 75 w/o FCS | 3.2·10^-1^ | 2.4·10^-1^ | 4.8·10^-2^-5.9·10^-1^ |
| 20+FCS | 1.0·10^-1^ | 1.9·10^-2^ | 7.3·10^-2^-1.3·10^-1^ |
| 50+FCS | 4.2·10^-2^ | 4.7·10^-2^ | -1.2·10^-2^-9.5·10^-2^ |
| 75+FCS | 1.7·10^-1^ | 1.3·10^-1^ | 2.7·10^-2^-3.2·10^-1^ |

Table S 2 Overview of the uptake and elimination rates based on AgNP numbers.

Average elimination rate constants k, their standard deviation (Std. dev) and 95 % confidence Interval (CI) based on AgNP numbers, all given in AgNPs·well-1·day-1.

| NP size [nm], medium type | k | Std. dev. of k | 95% CI of k |
| --- | --- | --- | --- |
| 20 w/o FCS | 1.4·10^-1^ | 4.3·10^-2^ | 8.4·10^-2^-2.0·10^-1^ |
| 50 w/o FCS | 8.6·10^-2^ | 7.6·10^-2^ | 4.2·10^-4^-1.7·10^-1^ |
| 75 w/o FCS | 3.0·10^-1^ | 2.3·10^-1^ | 3.44·10^-2^-5.6·10^-1^ |
| 20+FCS | 9.1·10^-2^ | 1.5·10^-2^ | 7.0·10^-2^-1.1·10^-1^ |
| 50+FCS | 3.7·10^-2^ | 4.1·10^-2^ | -9.3·10^-3^-8.4·10^-2^ |
| 75+FCS | 1.6·10^-1^ | 1.2·10^-1^ | 2.3·10^-2^-3.0·10^-1^ |
